# Evaluating an online support package delivered within a disability unemployment service: study protocol for a randomised controlled feasibility study

**DOI:** 10.1186/s40814-015-0010-6

**Published:** 2015-04-10

**Authors:** Carrie-Anne McClay, Stuart Rae, Jill Morrison, Alex McConnachie, Colin Maxwell, Christopher Williams

**Affiliations:** 1 Gartnavel Royal Hospital, Institute of Health and Wellbeing, University of Glasgow, 1st Floor Admin Building, 1055 Great Western Road, Glasgow, G12 0XH Scotland; 2 Mental Health Directorate, Cree West, Crichton Hall, Dumfries, DG1 4TG Scotland; 3 General Practice & Primary Care, Institute of Health and Wellbeing, University of Glasgow, 1 Horslethill Road, Glasgow, G12 9LX Scotland; 4 Robertson Centre for Biostatistics, Boyd Orr Building, University of Glasgow, Glasgow, G12 8QQ Scotland; 5 Remploy, 22-24 Earl Grey Street, Edinburgh, EH3 9BN Scotland

**Keywords:** Depression, cCBT, Guided self-help, Online, Unemployment, Bibliotherapy, Low mood, Stress, Anxiety, Disability

## Abstract

**Background:**

Mental health problems such as anxiety and depression are known to be higher in those who are unemployed. Cognitive behavioural therapy (CBT) is a recognised support for people with such problems and can improve the ability of people to get back to work.

**Methods/design:**

Participants with symptoms of low mood will be recruited from the disability employment service, Remploy. Participants will receive either immediate or delayed access to an online CBT-based life skills intervention, the “Living Life” package. The primary end point will be at 3 months when the delayed group will be offered the intervention. This feasibility study will test the trial design and assess recruitment, retention, acceptability and adherence, as well as providing efficacy data.

**Discussion:**

The study will inform the design and sample size for a future full randomised controlled trial (RCT) which will be carried out to determine the effectiveness of the online package in improving mood and employment status.

**Trial registration:**

Current Controlled Trials ISRCTN10316077.

## Background

Mental health problems such as anxiety, distress and depression are known to be more common in those who are unemployed [1], with lasting effects on mood in the long term, even after re-entering employment [2]. Unemployment rates are higher among those with disabilities, and therefore this may be a group that is vulnerable to experiencing poor mental health, particularly low mood. The National Institute for Health and Clinical Excellence (NICE) recommends cognitive behavioural therapy (CBT) for mild to moderate depression [3], and this treatment has an evidence base for use in back to work settings [4]. Traditional CBT consists of 12–20 1-h sessions with a mental health expert and can be delivered in primary care settings [5]. However, it is difficult to provide this *high-intensity* specialist CBT (HI CBT) due to the large volume of people with depression needing treatment. In order to increase access to CBT, low-intensity (LI) CBT in the form of bibliotherapy (online or paper based) can be successful in improving symptoms in low-risk individuals whose condition is not complex [6,7]. NICE [3] and Gellatly et al. [8] recommend that including guidance/support in using bibliotherapy and cCBT significantly improves outcomes for those with depression using these approaches. Crucially, the support does not need to be delivered by a mental health or CBT expert, and the focus can be on supportive monitoring. The current study aims to evaluate a novel online resource that encompasses online books, training modules, worksheets and reminder emails that can be used as a tool for those aiming to get back to work.

### Remploy

Remploy is the leading provider of disability employment services in the United Kingdom. It supports those with physical and/or complex problems that affect employment. Remploy offers training and support to 2,500 people per annum through its Scottish branches. Of these, around one in four will have experienced stress or low mood at some point. Currently, support is offered both directly and also by using referrals to other organisations. Usually individuals who are out of work are referred to Remploy by their local job centre or other organisation. The usual procedure when a new referral occurs is for an initial orientation meeting to take place. Candidates then receive weekly or fortnightly support from a case worker and they are supported into, and after they have entered, employment up to a maximum of 6 months post-work (and up to 2 years in-work support).

This will be the first study to evaluate cCBT self-help resources specifically for depression in those seeking employment delivered with LI support/guidance in this type of back to work environment. Our project draws on current NICE guidelines and offers the possibility of increased access to psychological therapy with the support of employment advisors. The “Living Life” online package will be delivered by Remploy and will include those currently attending the NHS as well as those who are not.

### The Living Life website

The Living Life online package contains eight core sessions that teach a range of CBT-based life skills [9]. The Living Life content is drawn from that of the successful and widely used www.llttf.com website but is delivered via a password-protected separate website containing the course plus linked reading and worksheet materials. The majority of the sessions will be completed in the Remploy offices; however, participants will have the option of completing and/or revising sessions on their home computer if they have one. Each session focuses on a different common problem faced by people when they feel low or anxious. The class content is the following: *1: Why do I feel so bad?, 2: I can’t be bothered doing anything, 3. Why does everything always go wrong?, 4: I’m not good enough: (low confidence), 5: How to fix almost everything, 6: The things you do that mess you up, 7: Are you strong enough to keep your temper?* and *8: 10 things you can do to help you feel happier straight away.* Additional physical health sessions are available including “Reclaim your life (from illness and disability)”, “Stop smoking in 5 minutes” and “Fix your drink problem in 2 days”. The package is designed to encourage an individualised plan to be made at the end of each session using a Plan, Do, Review structure [9]. Each session consists of slides, audio, worksheets and a linked booklet that summarises the topic and includes work tasks to facilitate practice at home. The package offers guided CBT for depression delivered over a series of online modules. There are currently no equivalent packages using the same model of delivery. This accessibility is reinforced by the language used. The resources use everyday terms and avoid professional terminology, i.e. the “life skills training” instead of “CBT” and “low mood and stress” rather than “depression and anxiety”. The way of communicating CBT used is highly accessible [10] and effective [11,12]. The language used in the online package includes everyday terms to describe symptoms such as stress, distress and low mood rather than the more formal diagnostic terms depressive disorder, depression and anxiety, etc. Although non-English language versions of the courses are available, the English language version is used in the current study to facilitate support by Remploy staff. The aim is to be inclusive and for the online resource to be appealing to participants who are being recruited through Remploy.

### Aims

This project aims to determine the feasibility of a randomised controlled trial (RCT) investigating an online CBT-based life skills resource for people who are seeking work and attending Remploy. It is an unfunded study to establish viability of applying for a substantive later funded RCT.

The aim of this study is to recruit people experiencing symptoms of depression. We will include both those with clinically diagnosed major depression as well as those with raised mood scores. We will describe the population in detail in terms of mood severity and clinical diagnosis (if known). The research questions of the study are outlined below.

### Research questions

Primary question:

- Is the study design feasible—is it possible to recruit from Remploy, randomise participants and collect data at baseline, 3 months and 6 months?

Secondary questions

b. To what extent will participants adhere to the intervention?
b. Is the Living Life package satisfactory/acceptable to participants?
c. How many participants will be needed for a sufficiently powered future RCT? (Efficacy data at 3 and 6 months will be used for the future sample size estimation).

## Methods/design

### Overview

This is a feasibility study to test the possibility of delivering the Living Life (LL) online life skills resource in Remploy. In this RCT, 50% of participants will be randomly allocated to the immediate group (IA) and will receive LL immediately. The remaining 50% of participants will be allocated to the delayed access control (DAC) group. Both arms receive treatment as usual. The primary end point is 3 months at which point the control arm will be provided with access to LL if they wish. Monitoring will continue up to a 6-month final exit point.

### Participants

The aim is to recruit 100 participants in total for this feasibility study, 50 intervention participants and 50 control participants to allow for comparison at the 3-month follow-up point and 6-month exit point. We will recruit males and females aged 16 +.

*Inclusion criteria:* Participants will be willing to take up the offer of LL and appropriate for usual Remploy support—and with no active risk to self at initial Remploy assessment (this is not routinely investigated, but if suicidality is disclosed at the initial assessment, the individual will not enter the study). Individuals must be able to register on the website, read and understand the LL resources via the internet or DVD; at home or in the Remploy office.

*Exclusion criteria:* Individuals will be excluded if they are unwilling or unable to use the LL approach and/or if they are at active risk of suicide, identified in which case usual care (referral to GP) will occur as is normal Remploy practice.

### Procedure for recruitment and study setting

All new candidates will be informed of the study, and their Remploy case loader (support worker) will provide a study information pack and consent form. The potential participant will be asked to bring the consent form to their meeting the following week if they wish to take part in the study. The researcher’s contact details will be on the information sheets to allow candidates to ask questions about the study (meaning the consent given will be informed consent). The Remploy staff can also answer any questions required. Taking up or declining the study will not affect the usual support received.

On attendance at the Remploy Office for the second appointment, completed consent forms will be collected by administration staff. Participants will then hand in their baseline questionnaires to the administration staff. The administration staff will mark up the participants’ case notes as being in the study and pass a treatment allocation envelope to the case loader. The sealed allocation envelopes will have been prepared remotely by the researcher and will be selected in the presence of the participant from a box to reveal the group allocation. Envelopes will state which course they have been enrolled in (1—immediate, 2—delayed). Those allocated to the LL group will be given details of how to access the online resources. Participants will work through the online modules in the Remploy offices either on their own or as part of a group with a trained Remploy facilitator, the delivery type for each participant will be recorded by Remploy. It will not be possible for the caseloader, participant or researcher to be blind to the group allocation.

All participants will receive supportive encouragement from a trained member of staff while they use the intervention. TAU participants will be given access to Living Life after the 3 month follow-up data has been collected. The “Living Life” intervention is password protected. However, in order to identify contamination at the 3-month follow-up, participants will be asked to detail any support they have received for their mood since entering the study.

### Ethical considerations

There are a number of ethical considerations that have been taken into account in the preparation of this protocol. These are as follows:

#### Recruiting adults only

Remploy only work with people who are older than 16 and in the work market.

#### Consent

After expressing an interest in the study, potential participants will be asked to complete an eligibility pack, which will include a participant information sheet along with a consent form. Participants will have the opportunity to ask questions about the study by phone, email or post before being asked to complete the consent form. Until eligibility has been assessed and informed consent has been given, individuals will not be able to begin participation in the study.

#### Confidentiality

Data collected by Remploy will be kept and stored in accordance with their usual internal procedures. Data collected by the research team will be kept either on fingerprint or password-locked memory sticks, on fingerprint identification laptops, stored in a locked filing cabinet in the researcher’s office on the site of Gartnavel Royal Hospital, or will be saved on a secure network space that only the researchers have access to. Personally identifying information will be removed from the main electronic data files for analysis by the researchers at the Robertson Centre for Biostatistics, University of Glasgow. A coded file with sensitive data will be stored separately.

### Baseline data collection

The standard information routinely gathered by Remploy will be used as baseline data. This will include demographic and employment information. In addition, mood and social functioning will be assessed via the Patient Health Questionnaire (PHQ-9—depression) [13], Generalised Anxiety Disorder Scale (GAD-7—anxiety) [14] and the Work and Social Adjustment Scale (WSAS—five-item social functioning scale) [15]. Current and recent medical treatment including medication will also be recorded.

### Follow-up data collection

The main follow-up point will be at 3 months with an additional follow-up assessment at 6 months. The data will be used to:

- Determine the proportion of people taking up the LL intervention, adherence to the intervention and follow-up rates.
- Describe the difference in proportion of people in work at 12 weeks between the LL group and the TAU control group or if not in work the number of interviews attended and job submissions made (measuring motivation).
- Describe the subsequent number (frequency) and total (days) off work over 3 months and 6 months in both arms comparing within group comparisons from baseline, and between-groups at 3 and 6 months.
- Describe and compare impact on mood, social function and satisfaction at 3 months and 6 months in both arms comparing within group comparisons from baseline, and between-groups at 3 and 6 months. This data will be used for the future sample size calculation.

### Measures

The following measures will be used in the study.

### Patient Health Questionnaire-9

PHQ-9 [13] is a freely available mood rating questionnaire consisting of nine questions mirroring DSM-IV depression diagnostic criteria and each rated 0–3 giving a maximum score of 27. Cut-off scores are used to label depression severity as:

- 0–4, minimal depression;
- 5–9, mild depression;
- 10–14, moderate depression;
- 15–19, moderately severe depression;
- 20–27, severe depression.

### Generalised Anxiety Disorder-7

The GAD-7 [14] is a seven-item questionnaire focusing on symptoms of anxiety experienced in the past 2 weeks. Each item is rated according to the frequency of the described problem. The responses are scored as follows: 0 = “not at all”, 1 = “several days”, 2 = “more than half the days”, 3 = “nearly every day”. Therefore, the maximum score is 21. Scores of 0–5 indicate mild anxiety, 6–10 = moderate anxiety, 11–15 moderately severe anxiety and 15–21 severe anxiety.

### Work and Social Adjustment Scale

The WSAS [15] will be used to assess levels of social functioning. The WSAS is a five-point questionnaire addressing life activities. The questionnaire probes issues relating to the impact the disorder in question is having on the individual’s everyday life and functioning. Responses are given on a scale of 0–8, with higher scores indicating higher level of impairment or disruption to social functioning. Therefore, the maximum score on this questionnaire is 40. Mundt, Marks, Shear and Greist [15] state that, in light of studies that included two clinical groups: in those with depression and those with OCD, a score below 10 on the WSAS does not indicate clinically significant problems and between 10 and 20 indicates significant impairment. Those scoring over 20 are likely to have significant problems in their social functioning [15].

### The Client Satisfaction Questionnaire

The Client Satisfaction Questionnaire (CSQ-8) will be administered post intervention as a measure of satisfaction [16]. The CSQ-8 is an eight-item questionnaire rated using a four-point Likert scale. Scores range from 8–32 with higher scores indicating greater satisfaction with the intervention in question.

### Statistical analyses

Baseline characteristics will be summarised for all participants as well as for the IA and DAC groups separately. Participant uptake and adherence to the online intervention, defined as the number of online sessions completed and the number of support sessions participants took part in, as well as data follow-up rates, will be summarised and presented as percentages.

To assess efficacy, repeated measures analysis will be carried out using an intention to treat approach by means of a linear mixed effects model with the time (baseline, 3 months, 6 months) and group (control, intervention) main effects and their interaction as fixed effects, and participant as a random effect. Models will be adjusted for age, gender and antidepressant use at baseline. Tukey post-hoc comparisons will be carried out with a familywise error rate of 5%. These models will be used to estimate and test the statistical significance of the within-group differences between time points and the between-group differences at each time point. Finally, a power calculation will be carried out to determine the required sample size for a future RCT.

## Discussion

### Importance of the project

Unemployment is a significant problem in the UK, with disabled people being more likely to be unemployed than those without a disability. The most recent labour force national statistics indicate that 45.6% of disabled people aged 16–64 was in employment in 2011, compared to 76.2% of working age adults without a disability. Those with a disability are also more likely to work fewer hours than those without a disability with around a third (33.8%) of disabled adults working part time hours [17].

Mental health problems such as depression are common in those with physical health problems and those who are unemployed [2]. cCBT interventions may help to improve mood, coupled with employment and health-specific modules, this intervention may improve mood as well as job-seeking and employment potential.

We hope that recruiting from Remploy will identify people who might otherwise fail to engage in traditional NHS-based services thus addressing the wider treatment gap. The design allows a detailed description of those recruited, in order to understand the age, current and past treatments, severity and chronicity of depression among those recruited.

If this study shows that the trial design is feasible, then this will lead to a larger, funded RCT of the intervention which may have important implications for supporting disabled people who are seeking to gain employment.

### Determining the feasibility of the study design

As outlined in the aims sections, the feasibility of the trial design will be determined by considering four main factors: recruitment, adherence, acceptability and follow-up rates. In the future RCT, the recruitment period is estimated to be 6 months, with the aim of recruiting approximately 100 people in total. Therefore, an acceptable recruitment rate in the current feasibility study is 3–4 participants per week for 6 months.

The adherence rate is defined as the number of online modules completed. We have examined completion rates of equivalent modules delivered in face to face classes. In our pilot study 65% of people attended half of face to face sessions [7]. We judge an acceptable completion rate as 55% of people completing four or more of the eight course modules. The number of support sessions participants take part in will also be used as an indicator of engagement and will provide information regarding the feasibility of the caseloaders taking on this role.

As discussed in the “Methods/design” section, we will be using the Client Satisfaction Questionnaire to assess acceptability of the intervention. Scores on this measure range from 8–32 with a median of 20. For this study scores ≥20 will be considered as acceptable.

Finally, in relation to the feasibility of collecting data, the main outcomes relating to employment are collected by Remploy as part of their standard procedures, so we expect to have approximately 100% follow-up rate for these variables (number of applications, employment status etc.) The remaining data concerning mood will be collected by the researcher. From our knowledge and experience of online trials, we believe a follow-up rate of ≥70% is acceptable for secondary outcomes; therefore, this follow-up rate would indicate feasibility of data collection.

### Benefits to participants

Participation within this research study will allow adults who are out of work and experiencing mild to moderate symptoms of low mood to use CBT-based educational online modules aimed at addressing these, and other, issues. Research has provided us with empirical evidence that the proposed approach is effective in aiding a variety of problems related to low mood and stress by teaching problem-solving skills, assertiveness, tackling negative thinking and increasing activity levels [6,7]. This study looks at applying similar approaches in a back to work setting.

Results from this research will be used to indicate whether the online intervention is an acceptable resource for those aiming to get back to work. By assessing the acceptance and feasibility of the package for this population, we will be able to gain a key insight as to how such an intervention can be best delivered to individuals within this type of organisation.

There should be few harms or discomforts as the online classes use everyday language and address widely experienced life skills’ problems. No formal diagnosis of depression or anxiety is made at any stage. Since the intervention is a life skills package that will be introduced as part of the Remploy service, we do not expect any emergencies. Participants will receive care as usual from Remploy, and any deterioration in their condition will be managed according to the usual Remploy guidelines. Professor Chris Williams and Professor Jill Morrison will also be available for consultation on such cases.

### Collaboration with Remploy—public engagement and knowledge exchange

Remploy are experienced in working with the proposed population, and their case workers will be able to assist any individuals who may need additional help. They are fully committed to helping with this study. Working with Remploy at this early feasibility stage will lay a foundation for the future large RCT which, if it is successful, will provide support for the introduction of cCBT within back to work organisations as an additional resource to offer to service users.

### Dissemination

Dissemination will include an executive summary, open access journal publications and conference presentations. The main journal publication will present the study findings with the data being used to inform the development of the full RCT protocol. A simple feedback sheet will be sent to all participants who have taken part. The findings will also be disseminated to Remploy management in the form of a report and an oral presentation. Remploy will then disseminate the results internally. We will also disseminate findings via the newsletters on the widely used www.llttf.com website.

## Trial status

Ethical approval has been granted by the College of Medical, Veterinary & Life Sciences Ethics Committee for Non Clinical Research Involving Human Subjects, University of Glasgow, Ref. 2012063. The case workers at the Remploy offices in Edinburgh and Hamilton have been briefed on the study protocol and recruitment process and have undergone training on how to deliver the support sessions to participants. Recruitment is ongoing; to date, 20 participants have entered the study.

## Abbreviations

CBT: Cognitive behavioural therapy
cCBT: Computerised Cognitive behavioural therapy
RCT: Randomised controlled trial
IA: Immediate access
DAC: Delayed access control
